# Reports on Cities, Towns, & Districts

**Published:** 1855-09

**Authors:** 


### SANITARY AND SOCIAL SCIENCE.

### REPORTS ON CITIES, TOWNS, & DISTRICTS.

### I.—ROMSEY.

THE town of Romsey, in Hampshire, has lately, at the request of Lord Palmerston, high-steward of Romsey, been subjected to a sanitary inspection by Dr. Waller Lewis. From his report the following is gathered.

Romsey possesses great sanitary capabilities: it has a protected situation, an abundant supply of clear rapidly running water, and very few manufactories. Its population was in 1841, 5347; in 1851, 5654; and in 1854, 5769; and the inhabited houses in 1841, 1096; in 1851, 1183; that is, about five persons to each house. The principal streets are tolerably wide, but many of the lanes and courts are close, narrow, and so filled with animal and vegetable refuse, ashes, and dirt, that it is "scarcely possible to name one street as more open to this reproach than another." A large proportion of the houses are small tenements and cottages, some being built back to back, and many having brick floors, of which that of the living-room is below the level of the street, and therefore always damp. The walls are also in many instances damp, filthy, and covered by a greenish moss. The rooms vary in height from six feet three inches to seven feet six inches, the back windows not being made to open, nor in some cases, even the front. One privy, also, sometimes accommodates sixteen or eighteen houses. The cesspools, nearly all mere excavations in the soil, are frequently imperfectly covered in, and, being not bricked or cemented, allow percolation to the pumps and wells, and have impregnated the greater part of the town soil. The practice common in the smaller streets and courts of keeping pigs also gives rise to effluvia, and furnishes additional matter for percolation. One woman, dwelling where this condition existed, had lost two out of six children by fever, and stated that the smell sometimes made them quite sick. The slaughterhouses are a continual source of complaint. The wells are generally fourteen or sixteen feet deep, insufficient in dry weather, and in wet, greatly charged with filth and fæcal matter. The wells near the churchyard are infected by its drainage into them. Numerous privies open into the brook, the water of which some persons prefer. Specimens of the water examined by Dr. Hassall contained infusoria, fungi, dead and decaying vegetable tissue, dirt, and grit. On standing for only a few minutes, they threw down a copious sediment.

The jail is unglazed, damp, and unfit for use; its privy is untrapped, and gives off foul gases into the male prisoners' room. The churchyard contains virtually but one acre, three roods, thirty-two poles, whereas there should be at least half an acre to every thousand inhabitants. Since 1659, more than 15,000 bodies have been there interred. The deaths during the years 1847 to 1853 were respectively, 124, 116, 193, 130, 127, 115, 121, in all, 926. The average mortality per 1000 during the last seven years has been 23·5, rather more than seven per 1000 being the average in the London establishments for the labouring classes. Dr. Lewis is of opinion that the necessary mortality is not more than from fourteen to sixteen annually; and says that Romsey contrasts favourably with other towns he has inspected in the desire of the labouring classes themselves for cleanliness. He suggests as remedies, a *constant supply* of pure water to every house, drainage, water-closets in place of privies, the annihilation of cesspools, and bringing the town under the operation of the Public Health Act; also, as palliatives, rooms not less than eight feet high, ground-floors raised above the street level, wooden floors, fitting dust-bins to each tenement, and lime washing internally and externally.

---

### II.—SANITARY STATE OF PARIS FROM THE COMMENCEMENT OF THE YEAR.

In the capital of France during the years 1853, 1854, 1855, the mean daily mortality in Paris has been,

| | 1853. | 1854. | 1855. |
|---|---|---|---|
| In January | 93 | 93 | 112 |
| February | 124 | 100 | 133 |
| March | 131 | 110 | 133 |
| April | 121 | 128 | 130 |
| May | 106 | 118 | 121 |
| June | 86 | 131 | 99 |
| July | 86 | 126 | 77 |

The mean mortality for the ten years, 1837 to 1848, has been,

| | | | | | |
|---|---|---|---|---|---|
| In January | 89 | In March | 96 | In May | 87 |
| February | 90 | April | 94 | June | 79 |
| In July | 74 | | | | |

Taking into consideration the increase of population, especially during the last four months, it will be seen that the average mortality of 77 for July 1855, is really considerably below the number, 74 representing the mortality in the ten years, 1837 to 1848. No sensible increase took place at the beginning of August. It follows, then, that the sanitary condition is the best possible, since with considerable increase of population, the average mortality does not increase, but on the contrary, remains lower than usual.

On other hand, it will be seen that the mortality of 1855 is much higher than the average in the months January to May: 112, 133, 133, 130, 121, instead of 89, 90, 96, 94, 87. This increase is principally due to typhoid fever, the intensity of which was very great during these months.

In the year 1853, a somewhat similar increase took place in the months of February, March, April, and May: 124, 131, 121, 106, in the place of 90, 96, 94, 87, To typhoid fever this also is due.

Again, in 1854, the cholera year, the mortality did not increase so much during the months of February and March, as during the corresponding months of the years without epidemic, 1853 and 1855; but the increase was rapid in the months of April, May, June and July. From these statistics it follows, that the season cannot be compared with that of 1854, nor any other cholera year. We have just gone through a season especially remarkable for its small number of diseases and low mortality.

It is important to compare, in this matter, the mortality of the second quarter of 1855 in England with the statistics just named for April, May, and June. The registrar-general's quarterly report shows that, during the second quarter of 1855, that the public health in England was in a great degree free from the pernicious influences of the last cholera epidemic and of a very severe winter. The annual mortality, which averages 22·57 per 1000 of the whole population, is only raised during the quarter to 22·79. The mortality of the rural districts, 20·88 per 1000, has been 20·97; that of towns, 24·50 per 1000, has not been higher than 25·03.

This comparison shows how general the influences are that act on public health. Examining the several diseases prevalent, we find that the number of febrile affections of intermittent or relapsing character has been very considerable; and this is one of the remarkable traits of the season just elapsed. More than that, a great number of ephemeral fevers exhibited at their commencement the general symptoms of typhoid. There were many cases with all the appearance of serious fever at their commencement; rosy spots showed themselves from the fifth to the sixth day, and before the tenth or twelfth day the fever abated, leaving only great weakness—in fact, a state of anæmia. Afterwards symptoms of scurvy often were developed. At other times typhoid fevers of moderate intensity, pneumonia, and a milder form of measles occurred, with tedious convalescence, irregular accessions of fever, persistent vertiginous headache, bleedings at the nose, and sharp pains in the legs. Some patients were the subjects of neuralgia. These various modifications of disease, which are rarely met with in ordinary practice, we have frequently seen; and we have found, in studying these facts, the distinctive characters of scurvy.—*Dr. Tholozan in the Gazette Médicale de Paris.*

---

### III.—NUISANCES IN ISLINGTON.

A TREMENDOUS and very proper outcry has lately been raised by some of the inhabitants of Islington against a nuisance which has for a long time past existed in Belle-isle, situated in York-road, and in the centre of a population numbering from 60,000 to 70,000 persons. The nuisance is caused by decomposing animal matter, and emanates from establishments covering at least eight acres of ground.

Whatever may be the opinions of physiologists as to the effects of bad smells in producing *special* diseases, there cannot be two opinions as to the propriety of removing so disgusting and of necessity so injurious a nuisance. The authorities of Islington seem, however to set themselves dead against all sanitary and social innovations. Not long since, Dr. Semple was turned out of his office as surgeon to St. Mary's workhouse, for having, with an honesty of purpose that will do him permanent honour, pointed out that the place was little better than a charnel house. The emanations from the Belle-isle nuisance seem unquestionably, from evidence which has been carefully collected, to have caused nausea and stomachic derangement amongst some of those who reside in the district.

---

